# Olfactory learning alters navigation strategies and behavioral variability in *c*. elegans

**Published:** 2023-11-13

**Authors:** Kevin S. Chen, Jonathan W. Pillow, Andrew M. Leifer

**Affiliations:** a Princeton Neuroscience Institute, Princeton;; b Department of Physics, Princeton, NJ, 08544

**Keywords:** olfactory learning, sensory navigation, statistical inference, *C*. elegans

## Abstract

Animals adjust their behavioral response to sensory input adaptively depending on past experiences. The flexible brain computation is crucial for survival and is of great interest in neuroscience. The nematode C. *elegans* modulates its navigation behavior depending on the association of odor butanone with food (appetitive training) or starvation (aversive training), and will then climb up the butanone gradient or ignore it, respectively. However, the exact change in navigation strategy in response to learning is still unknown. Here we study the learned odor navigation in worms by combining precise experimental measurement and a novel descriptive model of navigation. Our model consists of two known navigation strategies in worms: biased random walk and weathervaning. We infer weights on these strategies by applying the model to worm navigation trajectories and the exact odor concentration it experiences. Compared to naive worms, appetitive trained worms up-regulate the biased random walk strategy, and aversive trained worms down-regulate the weathervaning strategy. The statistical model provides prediction with > 90% accuracy of the past training condition given navigation data, which outperforms the classical chemotaxis metric. We find that the behavioral variability is altered by learning, such that worms are less variable after training compared to naive ones. The model further predicts the learning-dependent response and variability under optogenetic perturbation of the olfactory neuron AWC^ON^. Lastly, we investigate neural circuits downstream from AWC^ON^ that are differentially recruited for learned odor-guided navigation. Together, we provide a new paradigm to quantify flexible navigation algorithms and pinpoint the underlying neural substrates.

Learning is a fundamental property of the brain and is an important research topic in neuroscience. In order to study how the brain learns, the first essential step is to characterize the learning dependent behavioral outputs. One common approach to study learning in systems neuroscience is to train animals to perform relatively controllable but constrained tasks. While this approach leads to the discovery of certain neural mechanisms and learning rules (1, 2), there are growing evidence showing that learning is more prominent in naturalistic tasks (3, 4). Animals can learn naturalistic tasks in a few trials because it reflects the evolutionary pressure on the neural and behavioral algorithms (5, 6). Careful characterization of learned behavior in a naturalistic context sheds light on the flexible computation performed by the brain.

Olfactory navigation is a naturalistic behavior that is generic across species of different scales, has significant ethological relevance to detect long-range signal reflecting reward or aversion in the environment, and can often be acquired in few trials of learning. To study how animals learn to adaptively employ olfactory navigation strategies, we focus on the nematode worm C. *elegans* that has a compact nervous system and well-characterized olfactory neural circuits (7–9). The olfactory sensing mechanisms and navigation behavior in worms have been studied in detail (9,10). Despite the simplicity, it has been shown that past olfactory experiences lead worms to either seek out or avoid higher concentrations of odor (11–13). However, it is unknown how worms’ navigation strategies are altered by learning to achieve these feats. We aim to quantitatively characterize the learned navigation strategy, thereby constraining our understand of the underlying neural mechanisms.

Previous studies of navigation along salt and temperature gradients have revealed that the worm navigates in sensory environments mainly through two strategies: klinokinesis and klinotaxis (10, 14, 15). Klinokinesis corresponds to a process known as a biased random walk, in which the worm produces sharp turns called “pirouettes” with a probability that depends on local gradients (10, 16). Klinotaxis is a process in which the worm continuously modulates its heading to align with the local gradient. In worms, this process is also known as “weathervaning”, as the animal biases its head direction in response to local gradients (14). Past literature has found contributions from both strategies in a variety of sensory-guided navigation behavior, from temperature (15), and salt (14, 17, 18), to certain odor gradients (9, 14, 19, 20). In contrast to salt chemotaxis or thermotaxis (21, 22), airborne odor-guided navigation in the worm has not yet been captured by well-constrained quantitative models, in part because most experimental assays do not provide detailed information about the odor concentration experienced by the animal as it moves in the environment. Newly developed experimental setup to control and monitor the odor landscape experienced by the worm (23) now make it possible to empirically constrain models of odor guided navigation.

Here we focus on learned odor navigation in worms, which is particularly interesting because it involves altering the “valance” of an external stimulus through training. This is closer to forms of associative learning commonly studied in other animals, including mammals. In comparison, other forms of experience-dependent changes to behavior, such as salt chemotaxis and thermotaxis, are altered by the specific temperature or salt concentration for cultivation and forms a preferred “set point” memory for the environment the worm experiences in the past (15, 17). The fact that olfactory associations can be rapidly and flexibly learned has ethologically relevance, such as the ability to avoiding pathological food source from past aversive experiences (13, 24). Rapid odor learning enables the investigation of flexible neural computation and behavioral algorithms.

Butanone provides an ideal odorant for the study of associative learning. Past work has characterized the sensory neurons sensing butanone and demonstrated associative learning paradigms using butanone (11, 12, 25, 26). Butanone is a volatile organic compound that can be found in bacterial food in natural habitats (27), so worms are intrinsically attracted to the odor. When exposed to butanone paired with food (“appetitive training”), the worm increases its preferences to move towards the butanone source (8, 11, 12, 26). In contrast, when butanone is paired with starvation (“aversive training”), worms decrease their tendency to climb up butanone gradients in comparison to worms without exposure to butanone (“naive”) (11, 26). The chemotaxis index, defined as the fraction of worms that move towards the butanone source in a chemotaxis assay, is a classic measure for quantifying the effect of a learned association between butanone and the presence or absence of food. Key molecules and genes related to learning have been identified using this approach (12, 26). Recent work also analyze how the neural responses to odor impulse are altered by butanone learning (8). However, precise behavioral strategies that the worm alters upon learned odor-navigation remain unknown.

In this study we seek to answer: (1) How are the worm’s navigation strategies altered by olfactory learning? (2) How do the learned strategies vary across the population? (3) What neural substrates may be involved in learned odor navigation? To answer these questions, we combine precise experimental measurements and a novel statistical model to rigorously characterize how butanone associative learning alters odor navigation strategies in worms.

We investigate the worm’s odor navigation by measuring the locomotion in a controlled butanone odor environment and use those measurements to tightly constrain a novel generative statistical model that characterizes behavioral strategies in worms. By fitting the model to worm navigation trajectories after different training experiences, we find that the behavioral strategies are bidirectionally altered by butanone learning. We characterize the sensorimotor transformation from odor to navigation behavior by fitting the statistical model to data. The inferred model parameters are interpretable, better decode training conditions compared to chemotaxis index, and can also predict response to optogenetic perturbation in the sensory neuron AWC^ON^. We discover that naive worms have higher behavioral variability and demonstrate context dependent behavior. We also find that bidirectional learning effects is dominated by the AWC^ON^ sensory neuron pathway. Lastly, we genetically down regulate individual interneurons and find distributed contribution on behavioral strategies that are differentially affected by learning.

## Results

### Learning bidirectionally alters olfactory navigation.

**Fig. 1.**
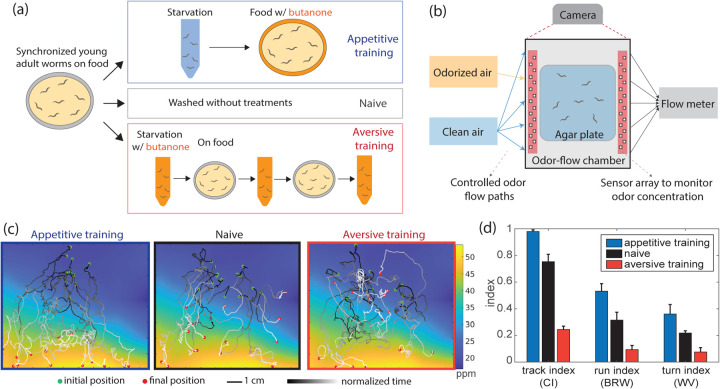
Bidirectional olfactory learning in C. elegans. **(a)** Protocol for butanone associative training in worms. **(b)** After exposure to different training regimens in (a), worms’ olfactory navigation is measured in a controlled odor environment. **(c)** Example trajectory after three different training conditions. **(d)** Summary statistics of learning across three conditions. The track index is the normalized number of tracks going up gradient $N_{u}$ versus down $N_{d}:\frac{N_{u}-N_{d}}{N_{u}+N_{d}}$, which corresponds to the chemotaxis index (Cl). The run index is the normalized run length going up gradient $r_{u}$ versus down $r_{d}:\frac{r_{u}-r_{d}}{r_{u}+r_{d}}$, which quantifies biased random walk (BRW). The turn index is the normalized probability turning up gradient $p_{u}$ versus down $p_{d}:\frac{p_{u}-p_{d}}{p_{u}+p_{d}}$, which quantifies weathervaning (WV). Error bar shows standard error of mean across 9–13 plates for each condition.

We developed a protocol to train worms to associate butanone with either food (appetitive training) or starvation (aversive training) (Figure 1a). Our protocol was similar to previously reported training regimens (8, 12, 26) except it exposes the animal to multiple rounds of odor paired with starvation instead of one, which increases consistency in learning (Fig. SI2). After training, the movement of populations of worms was recorded in a flow chamber with a known odor landscape (Figure 1b, Fig. SI1). We recorded hundreds of locomotory trajectories in this odor environment after different training conditions, per plate, for up to 13 plates per training condition. Qualitatively, the trajectories of appetitive trained worms are biased to travel up the butanone gradient; the trajectories of naive worms are less biased; and aversive trained worms appear qualitatively indifferent to the gradient (Figure 1c). We quantify the performance of traveling up the gradient using a chemotaxis index that is calculated from the animal’s trajectory (17). Our measurements show that animal performance navigating up gradient is bidirectionally modulated by learning, where chemotaxis index is up-regulated after appetitive training and down-regulated after aversive training compared to naive condition (Figure 1d). Interestingly, even animals that undergo aversive training do not navigate down the gradient, suggesting that after learning an association between butanone and starvation, animal are indifferent or still slightly attracted to butanone.

To quantify how much the worms biases the movement during a biased random walk, we calculated a run index by computing the normalized run length moving up-gradient. To compute how much the worms turns up gradient like a weathervane, we calculated a turn index by computing the normalized fraction of turns heading up-gradient (17). Our measurements show that both the run index and the turn index are on average bidirectionally modulated by learning with respect to naive animals, suggesting that learning alters both of these navigational strategies.

While these indices suggest hypothesis about how navigational strategies change due to learning, they provide little information about the dynamics during sensorimotor transformation. Specifically, the metrics use only binary information about whether the animal is traveling up or down gradient, but ignore details of the sensory landscape like the concentration experienced by the animal over time. The indices also provide no information about the behavioral stochasticity and variability across the population. To overcome these limitations, we sought to build a statistical model that captures temporal information, behavioral noise, and explicitly reveals how the animal changes its movement in response to sensory stimuli. The resulting framework better characterizes navigational strategies by incorporating the sensorimotor transformation from odor input to behavioral output in worms. This enables detail comparison for changes upon olfactory learning and makes quantitative prediction about behavioral response to sensory perturbation.

### Odor-dependent mixture model of olfactory navigation.

**Fig. 2.**
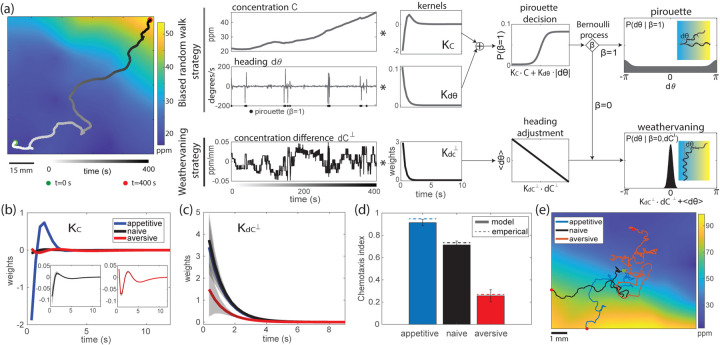
dPAW captures learned olfactory navigation in worms. **(a)** Schematic of the dPAW model. An example data trajectory is shown on the far left, providing time series of concentration and angle changes. Response kernels and decision functions are fitted to the time series data. The Bernoulli process β leads to two parallel strategies: biased random walk and weathervaning. **(b)** Kernel $K_{C}$ and **(c)** Kernel $K_{dC}⊥$ fitted to three training conditions. Shaded area shows standard deviation of the kernel estimate. **(d)** Chemotaxis index of simulated trajectories with inferred parameters. Error bar shows standard error of mean across 10 repeated simulation, each with 100 trajectories. **(e)** Example trajectories simulate from each training conditions.

To understand the how sensory inputs are transformed to behavior under different training conditions, we developed a new statistical model of worm olfactory navigation. The dynamic Pirouette and Weathervaning (dPAW) model consists a mixture of two navigation strategies — biased random walk through pirouette behavior and weathervaning—and describes how the worm alternates between two behavioral strategies depending on the time-varying sensory inputs (Figure 2a). dPAW explicitly models these strategies and fits to the full chemotaxis data. This is to our knowledge the first rigorous statistical model that captures the details of navigation strategies, which can better characterize how the worm’s behavior changes with learning.

In our model, the worm samples its heading change dθ at each time step from one of two distributions: either a “weathervaning distribution” $P_{wv}(d\theta )$ or a “pirouette distribution” $P_{pr}(d\theta )$. The weathervaning distribution is narrow, reflecting small changes in heading angle that result from the worm’s recent measurements of the concentration gradient. Pirouette behavior, on the other hand, corresponds to large turns that the worm makes when it receives evidence that it is going in the wrong direction. The pirouette distribution is therefore broad, with a peak at ±π, indicating a complete reversal in direction. The concentration-dependent “decision” to produce pirouettes behavior forms a biased random walk.

We model the worm’s decision to pirouette or weathervane on each time step with a Bernoulli generalized linear model (GLM) that takes the filtered history of the odor concentration $C_{1:t-1}$ and the worm’s own movement history $d\theta_{1:t-1}$ as inputs. The output of this GLM is a binary variable $\beta_{t}$ that indicates the presence of a pirouette. Thus, the worm samples its heading change from the pirouette distribution $P_{pr}(d\theta )$ if $\beta_{t}=1$ and the weathervaning distribution $P_{wv}(d\theta )$ if $\beta_{t}=0$. The full model can be written:

[1] $$P\left(d\theta_{t}|C_{1:t-1},dC_{1:t-1}^{⊥},d\theta_{1:t-1}\right)=P\left(\beta_{t}=1\right)P_{pr}\left(d\theta \right)+P\left(\beta_{t}=0\right)P_{wv}\left(d\theta |dC_{1:t-1}^{⊥}\right),$$

where

[2] $$P\left(\beta_{t}=1\right)=m+\frac{M-m}{1+\text{exp}\left(K_{C}\cdot C_{1:t-1}+K_{h}\cdot \left|d\theta_{1:t-1}\right|\right)}$$

is the mixing probability over the two distributions, with parameters m and M for the minimum and maximum probability of a pirouette on a single time bin, and $K_{C}$ and $K_{h}$ corresponding to filters on past odor concentration $C_{1:t-1}$ and the past absolute angular change $\left|d\theta_{1:t-1}\right|$ vectors, respectively. The pirouette and weathervaning distributions are in turn given by

[3] $$P_{pr}\left(d\theta \right)=\alpha U\left[-\pi ,\pi \right]+\left(1-\alpha \right)f\left(\pi ,\kappa_{pr}\right),$$

[4] $$P_{wv}\left(d\theta |dC_{1:t-1}^{⊥}\right)=f\left(-K_{dC^{⊥}}\cdot dC_{1:t-1}^{⊥},\kappa_{wv}\right)$$

where U is uniform in the circular heading and f is a von Mises distribution with mean and precision parameter κ. In the pirouette distribution, scalar α∈[0,1] is the weight on the uniform distribution and $\kappa_{pr}$ is the precision parameter that determines the sharpness of the pirouette. In the weathervaning distribution, the mean is altered according to the perpendicular concentration change $dC_{1:t-1}^{⊥}$ and the precision parameter $\kappa_{wv}$ determines the noise around the head angle.

We fit dPAW to the experimental measurements, including the time varying headings $d\theta_{t}$, the concentration along the locomotion path $C_{t}$, and the concentration perpendicular to the locomotion path $dC_{t}^{⊥}$. All model parameters in dPAW are jointly inferred through maximum-likelihood method. To validate that parameters can be reliably inferred, we simulated example chemotaxis trajectories from pre-defined parameters, fit them to the model, and confirmed that we accurately recovered the parameters (Fig. SI3). In the rest of the paper we use this model to interpret how navigation strategies change with learning, to quantify variability across behavioral trajectories, and to decode the animal’s past experience.

### Model captures navigation strategies altered by olfactory learning.

We seek to characterize how olfactory learning alter navigation strategies. We fit dPAW to trajectories measured in the butanone odor environment after different training conditions. The result shows that the kernels corresponding to both weathervaning and biased random walk strategies are altered by learning (Figure. 2b,c). The weathervaning kernel $K_{dC_{}^{⊥}}$ has lower weights after aversive training compared to appetitive trained or naive animals (Figure 2c), suggesting that the animal’s heading angle is less tightly dependent upon the concentration difference perpendicular to its path. In the kernel for pirouette decision $K_{C}$, the amplitude is significantly increased after appetitive training compared to naive animals, suggesting that the animal’s pirouette probability strongly depends on the concentration change along the navigation path. After aversive training, the kernel $K_{C}$ has a longer time delay and forms a tri-phasic shape, markedly different from the biphasic shape observed in naive animals, suggesting that aversive trained animals may not be responding as much to downward changes in concentration (Figure 2b). Collectively, these changes in the sensing kernels provide insights into how the worms flexibly alters their sensorimotor transformations locally to achieve the observed learned chemotaxis (Figure 1d). This precise effect on the way worms compute local odor concentration is not apparent from an analysis of chemotaxis index alone.

To validate that dPAW captures biologically plausible navigation behavior, we use it to simulate chemotaxis behavior with the fitted parameters from animals in the same odor environment previously exposed to three training conditions. dPAW with fitted parameters produces macroscopic behavior similar to the measurements (Figure 2d). Similar to the measurements, the model-generated behavior has a chemotaxis index that is bidirectionally modulated by training conditions. Example model-generated chemotaxis trajectories also appear similar to experimental observations (Figure 2e). We show that the simulated trajectories capture other statistics measured in experiments, including the heading distribution, pirouette rate, perpendicular concentration difference, and the tangential concentration distributions (Fig SI 4,5).

**Fig. 3.**
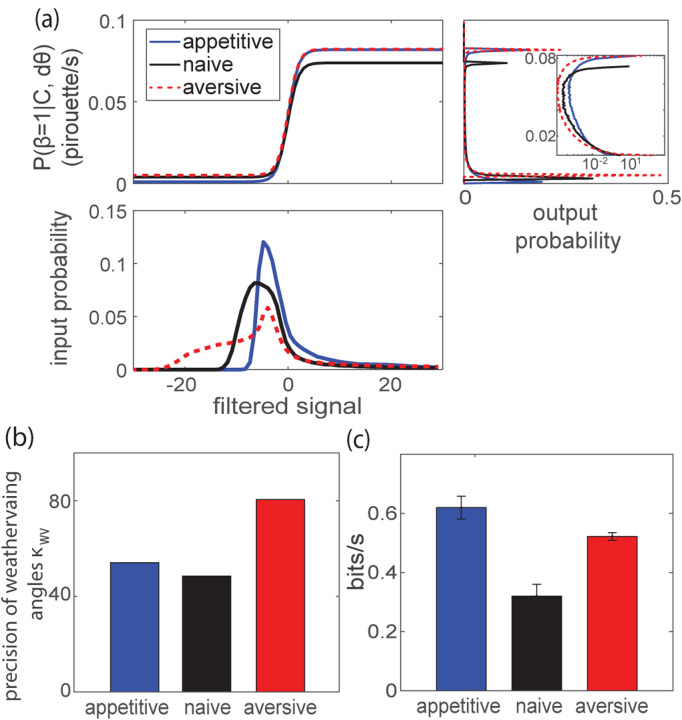
Learning alters pirouette decision and and behavioral noise. **(a)** The top panel shows decision function P(β=1∣C,dθ) of three training conditions as a function of filtered signal: $K_{C}\cdot C+K_{h}\cdot |d\theta |$. The distribution of the filtered signal is shown in the bottom panel. The right panel shows the distribution of the output pirouette rate, with inset showing the same distribution in log scale. **(b)** The precision parameter for weathervaning, $\kappa_{wv}$ across three training conditions. **(c)** The information rate given fitted model parameters and data across three training conditions. Error bar shows standard error of mean across 10 sampled batches of trajectories.

To understand how the animal alters its preference for continuing to weathervane versus interrupting weathervaning with pirouettes, we compared the decision function in equation 2 inferred from our measurements before and after learning. We found that learning alters the input-output statistics for producing the pirouette behavior (Figure 3a, bottom). This is clear from inspecting the distribution of the filtered signal (related to odor concentration and past behavior), where appetitive training and aversive training pushes the tail of the distribution in opposite directions with respect to naive condition.

Passing the filtered signal (Figure 3a, bottom) through the nonlinear decision function (equation 2 and Figure 3a, top), we find that aversive trained worms have higher baseline turning rate m and appetitive trained worms have a lower one (Figure 3a, right). Both aversive and appetitive trained worms have higher maximum pirouette rate M compared to naive worms. The parameters M and m are not simply reflecting the overall pirouette rate. The fitted model parameters “explain” the pirouette behavior differently – an observed pirouette behavior is more likely to be spontaneous for aversive trained worms and is more likely to be an odor-driven event in appetitive trained worms. Inspecting these output distributions in a log scale (Fig 3a right, inset), it is interesting to note that appetitive (but not aversive) trained animals spend more time with their pirouette probabilities in the most sensitive range of the sigmoid, possibly indicating a more efficient strategy for chemotaxis.

To confirm that the model accurately captures the empirical pirouette frequency, we compared model predictions and the classic threshold-crossing method to detect sharp turns in experimental measurements (10). The model estimation agrees with empirical pirouette rate across training conditions (Fig. SI4). Together, the result shows that dPAW captures the pirouette decision and reveals that learning alters both the processing of sensory input and the probabilistic behavioral output. The model also helps us understand how pirouettes are more or less often odor-driven under different training conditions.

Analysis of the kernel amplitude reveals that weathervaning was down-regulated after aversive training (Figure 2c). One hypothesis for a disrupted weathervaning behavior is that the worm is uncoordinated and randomizes its heading direction more during weathervaning, such that the weathervaning process itself becomes more similar to a continuous random walk. We show that this is not the case. On the contrary, the worm’s trajectory after aversive training has a longer persistence length. This is shown from the inferred precision parameter for the weathervaning process $\kappa_{wv}$, which is larger after aversive training (Figure 3b).

We further investigate the functional contribution of two strategies with dPAW by investigating two types of alternative models that omit key information. In the first, we set the two sensing kernels separately to zero and find that the $K_{C}$ that affects the pirouette decision is highly informative across all training conditions (Fig. SI5a). In the second type of null model we remove odor dependence entirely, but preserve the average pirouette rate. dPAW with the concentration-dependent strategies on average provides 0.3 – 0.6bits/s more information compared to the null model without any olfactory sensing mechanisms (Figure 3c).

### Model-based decoding of learned experience.

**Fig. 4.**
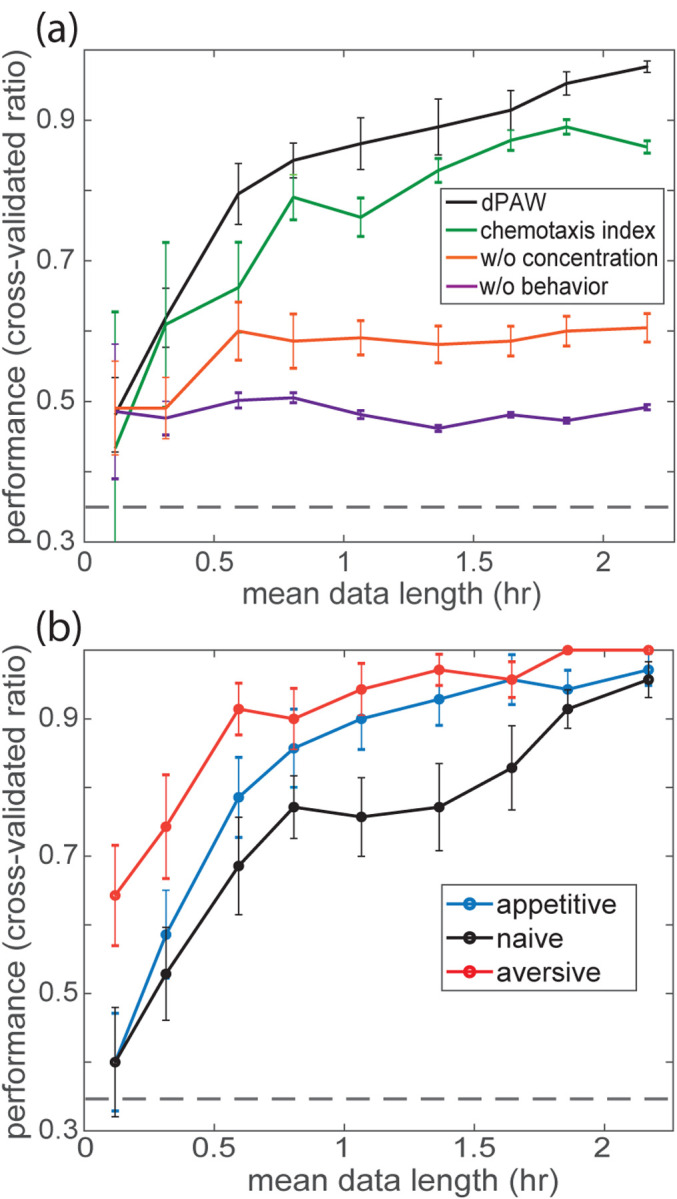
Model-based decoding of learning. **(a)** Model-based classification as a function of mean data length. Four models are compared and error bars show standard error of mean across 7-fold cross validation and 10-fold sampling across data length. w/o concentration: model that only captures behavioral statistics and does not account for odor input. w/o behavior: model for concentration difference along each trajectory and does not account for behavioral output. Chance level is shown in grey dash line. b dPAW-based decoding as a function of data length, for each training conditions calculated separately.

How much do the worms’ navigation strategies differ after learning? To address this question, we performed model-based decoding of the past training experiences. We first fit separate dPAW models to measured trajectories from each training conditions γ∈{appetitive, naive, aversive } giving the corresponding fitting parameters $\Theta_{\gamma }$. Then we make maximum likelihood predictions of the condition given held-out test trajectories: ${argmax}_{\gamma }P\left(\vec{d\theta }|\vec{C},d\vec{C}{\vec{C}}^{⊥};\Theta_{\gamma }\right)$. On held out data, the fitted model correctly predicts past training condition with a performance above 90%, which is significantly above the 33.3% chance level (Figure 4a). We compare the performance with a classical chemotaxis metric – the fraction of tracks that go up gradient. This metric can be formulated as a binomial model that estimates the probability of observing tracks going up gradient. The chemotaxis metric reaches only 80% accuracy at decoding and is continuously outperformed by our dPAW model for finite testing data.

Why might dPAW perform better? dPAW derives its power in part from using the animal’s combined concentration experience and behavior history, what we call its navigation trajectory. Together, this contains more information than, for example, the binary outcome of up or down gradient. We found that predicting learning experiences from concentration alone – without behavioral information – performs worse, suggesting that its not the full concentration history alone that trivially provides all the needed information (Figure 4a). For instance, the distribution of concentration change along all tracks in each condition is not separable enough to reach 90% predictive performance (Fig. SI7). Even if we take the concentration trajectory and compute the difference of concentration in the beginning and ending of each trajectory, the predictive performance is only around 50%. This suggests that combining moment-by-moment concentration information with behavior information is necessary for optimal decoding.

Similarly, dPAW also outperforms models or metrics that rely only on behavior and without the animal’s experienced concentration. When considering a model that only captures the behavioral statistics but neglects the odor concentration, the predictive performance plateaus around the 60% performance. We note that because this is above chance, it indicates that learning alters the overall behavioral statistics as well. In other words, we demonstrate that even without knowing any aspect of the sensory environment, simply observing the statistics of motion provides information about the animals learning. Together, the results show that the detailed sensorimotor transformation between odor concentration and navigation behavior is important for decoding. The learned parameters in dPAW better characterizes how the worms learned to navigate and can generalize to make predictions on trajectories measured in new experiments.

Model-based prediction characterizes how well the fitted parameter generalize to new test data. Given variable behavioral data, such analysis can guide experimenter to repeat more variable conditions to gain better estimates of the behavioral strategies. Specifically, trajectories from naive worms show the lowest predictive performance with finite data when decoding is done for each training conditions separately (Figure 4b). This is consistent with lower information rate and variable parameter estimates shown in Figure 2b and Figure 3c. Empirically, this result indicates that more measurements are required to capture navigation behavior in naive worms.

### The same computations govern natural and artificial odor stimuli.

We investigated response to optogenetic induced odor sensation after olfactory learning. The motivation for using optogenetics is to probe casual sensory motor responses that are not confounded by the correlations in the odor stimuli experienced by the animal in a naturalistic environment. For example, the worms in our arena always experience temporally correlated and slowly varying odor responses, but with optogenetics we can probe the animal’s response to arbitrary stimulation, including impulses (26, 28). We wondered whether the underlying computations that govern the animals response to natural odor stimuli were similar to those governing its response to optogenetic-induced sensory stimuli. We therefore included optogenetic perturbation in the experiments and adopted our model to incorporate such stimuli.

**Fig. 5.**
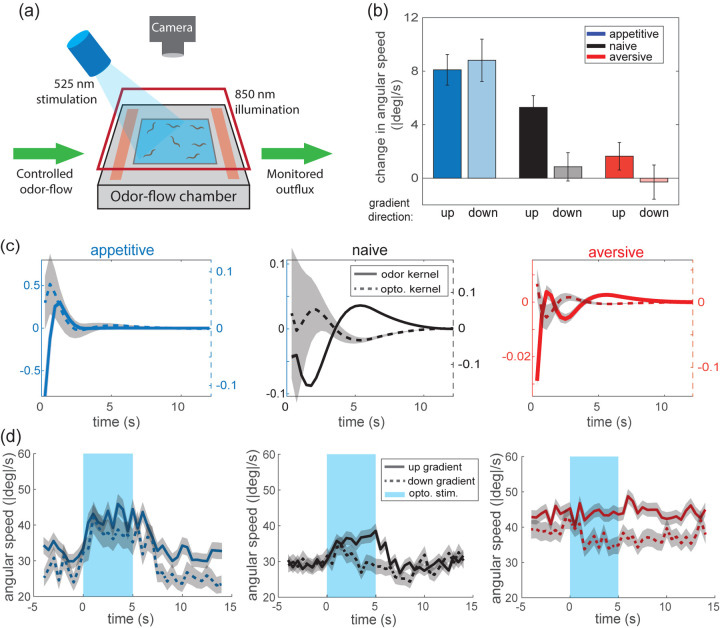
Optogenetic perturbation in the context of learned odor navigation. **(a)** Experimental setup that combines optogenetic perturbation with the odor-flow chamber. **(b)** Absolute change in angular speed upon optogenetic impulse across thee training conditions. Change is computed between the average response during the 5 s impulse and 5 s before it. Within each training conditions, measurements are further separated into trajectories going up or down gradient. We record from 4–7 plates per condition. Error bar shows standard error of mean across over 1000 impulses per condition. **(c)** Temporal kernels for odor $K_{C}$ and optogenetic $K_{\text{opto}\;}$ fitted to each training conditions. The grey area shows standard deviation around the estimated kernels. **(d)** Time-varying absolute angular speed aligned to the optogenetic stimuli. Three panels show different training conditions. Solid line and dash line indicate up and sown gradient trajectories. The grey area shows standard error of mean across traces.

We presented optogenetic stimulation to worms expressing channelrhodopsin in AWC^ON^, a sensory neuron known to sense butanone and play an important role in learning (8, 12, 26). AWC^ON^ was optogenetically stimulated in worms as they navigated in the odor arena, therefore they received both optogenetic-induced and natural odor stimuli simultaneously (Figure 5a). The absolute angular change in response to an optogenetic impulse was bidirectionally modulated by learning. This is similar to results shown in (26), but note that here we use the exact same naive control to compare with appetitive and aversive training conditions (Figure 5b,c). We extend our model so that light intensity contributes to the biased random walk strategy and the pirouette probability during navigation:

[5] $$P\left(\beta_{t}=1\right)=\frac{M-m}{1+\text{exp}\left(K_{\text{odor}\;}\cdot C_{1:t-1}+K_{\text{opto}\;}\cdot I_{1:t-1}\right)}+m$$

where kernels $K_{\text{odor}\;}$ and $K_{\text{opto}\;}$ are weights on vectors of odor concentration $C_{1:t-1}$ and light intensity $I_{1:t-1}$, respectively. Since there is no difference along the tangential direction in optogenetic signal, this model is simplified with kernels weighting the tangential concentration and neglecting the perpendicular concentration for weathervaning. Surprisingly, across different training conditions, the optical kernel is close to the mirror image of the odor kernel, most strikingly demonstrated in the naive and aversive conditions (Figure 5d; Fig. SI8). Our result provides empirical evidence that sensorimotor computation inferred from freely moving animals can predict the response to external perturbations. The inversion can partly be explained by the biophysics of AWC neuron: it hyperpolarizes when odor is present and depolarizes when odor is removed, therefore we expect optogenetic stimulation to have an opposite effect as odor presentation. This gives us confidence that our findings about sensorimotor processing derived from natural odor stimuli should be relevant to the larger literature based on more artificial stimulation. Similarly, we can leverage optogenetics to probe behavioral variability during odor navigation.

### Learning modulates behavioral variability in response to sensory perturbation.

Sensory processing and navigation behavior can be intrinsically noisy in worms (29). We first sought to characterize the variable navigation behavior across the worm population by analyzing sub-sampled tracks during natural odor stimuli (Fig. SI6). We found that the biased random walk kernel $K_{C}$ in naive and aversive trained conditions are more variable across ensembles. To better identify the source of the variability, we again turn to an optogenetic approach to probe behavioral response in the context of navigation and learning.

Surprisingly, we discovered that one source of variability was the context of gradient direction. We found that naive and aversive trained worms respond to optogenetic impulse differently depending on whether the navigation trajectory is going up or down gradient (Figure 5b). Appetitive trained worms respond consistently to optogenetic impulse regardless to the context of gradient direction, whereas naive and aversive trained worms respond more strongly in the context of moving up gradient. This indicates that learning alters behavioral variability, which in turn can be context-dependent along the navigation trajectory. The result that naive worms exhibit more variability in their odor guided navigation is consistent across three different analyses: (1) naive worm produce trajectories that have less bits per seconds captured by the model (Figure 2b and 3e), (2) the decoding performance of naive worms is lower with finite data (Figure 4b), and (3) naive worms’ response to optogenetic stimulation is more variable (Figure 5d) and context-dependent (Figure 5b). Together, this shows that naive worms have more heterogeneous navigation strategies and sensorimotor responses. And at least some of that variability can be explained by its context-dependent interpretation of odor stimuli, depending on whether it travels up or down gradient.

### Downstream interneurons deferentially contribute to learned chemotaxis.

**Fig. 6.**
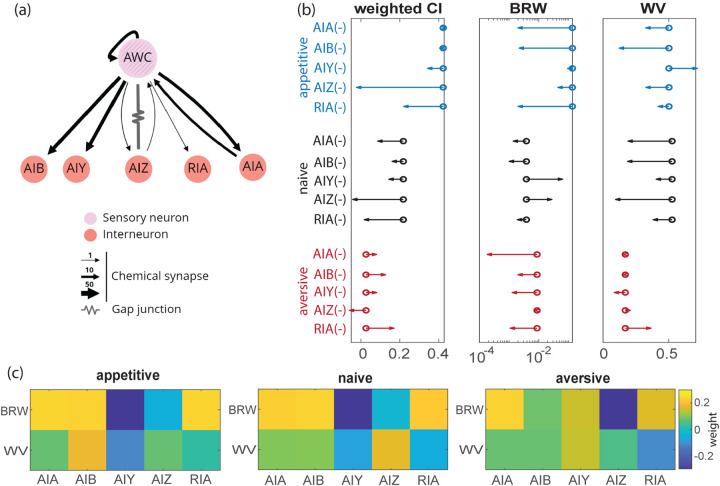
Learned odor navigation in worms with disrupted interneurons. **(a)** Five interneurons connected to the AWC sensory neuron. Schematic generated from nemanode.org **(b)** Weighted chemotaxis index (Cl), biased-random walk (BRW), and weathervaning (WV) indices computed from the inferred dPAW for all mutants across three training conditions. The circles show value from wild-type N2 worms and arrow shows the modulation in mutant worms. We record 3–5 plates for each stain and condition, resulting in 200–500 tracks in each measurement. **(c)** Weights in the neuron-behavior matrix W across three training conditions. The two rows are for different behavioral strategies and the columns are for different mutant worms.

Measured behavior under optogenetic stimulation shows that driving AWC^ON^ neuron is sufficient to recapitulate the effects of bidirectional learning. Past work has also shown the importance of AWC^ON^ in terms of odor sensing (9), navigation (30–32), and learning (11, 26). We further investigated the downstream circuit that implements this sensorimotor computation by ablating or down-regulating specific interneurons in worms, and then characterized defects in the learned odor navigation using dPAW. Following known anatomical connections (7) (Figure 6a), we focused on five interneurons with direct synaptic inputs (chemical or electrical connections) from AWC: AIB, AIZ, AIY, RIA, and AIA.

We compared the chemotaxis performance and behavioral strategies in mutants to wild-type worms, and conducted significance test with sub-sampled data (Figure 6b; Fig. SI9, Fig. SI10). Without learning, naive worms with individual disrupted interneurons can still climb up gradient, except for AIZ(−) worms. AIZ(−) condition eliminates gradient climbing behavior across all three training conditions. RIA(−) worms have down-regulated chemotaxis indices for both appetitive and naive conditions, but show increased chemotaxis index in aversive conditions. This indicates that in RIA(−) defective mutants there is no bidirectional change to navigation upon learning. AIA(−), AIB(−), and AIY(−) worms have less difference between naive and aversive training conditions, but still preserve up-regulated chemotaxis performance after appetitive training conditions.

We systematically quantified the effects of interneuron perturbation with a linear model (Figure 6c): B=WA, where B is the matrix with behavior readout along the rows and ablation conditions along the columns, W is an unknown weight matrix from neuron to behavior, and A is the ablation matrix with binary elements. In our experimental design, if we assume that each transgenic line is specific enough to perturb one interneuron at a time, the diagonal elements would be zero and all off-diagonal elements are one. This matrix is full-rank and makes the linear model solvable. We normalized the rows in B to better compare weights across behaviors.

We studied W (Figure 2c) and found that AIA contributes strongly to biased random walk in aversive trained conditions, which agrees with observation in (26) showing that AIA is required for aversive learning and alters turning responses. AIB and AIY contribute to both behavioral strategies (31), but the contribution differs depending on the training conditions: the contribution of AIB is enhanced for appetitive condition and the contribution of AIY is enhanced for aversive condition. AIZ contributes to weathervaning in all conditions and is significantly higher in naive condition. Our result is consistent with the finding showing that ablating AIZ destroys salt chemotaxis behavior reported in (14). Lastly, RIA has negative weights in the aversive condition, possibly reflecting the importance of regulating weathervaning in the aversive condition shown in Figure 2c, and in agreement with RIA’s known role in controlling head motion (33). Overall, the weight matrices W are more similar between appetitive and naive conditions than aversive. Our analysis shows that the contribution on behavioral strategies are distributed across interneurons for all training conditions.

## Discussion

We combined precise experimental measurements and a novel statistical model, dPAW, to characterize learned olfactory navigation in worms. The results show that (1) navigation strategies are bidirectionally altered by learning, (2) dPAW decodes the animal’s past training conditions based on the observed navigation behavior and outperforms a classic chemotaxis metric, (3) behavior is more variable in naive worms and some of this variability can be explained by a contextdependent response to odor, and (4) interneurons downstream from AWC^ON^ contribute to learned navigation strategies differently depending on learning.

Worms respond to a range of sensory input in a experiencedependent manner (17, 34). Previous studies show how neural responses are altered by learning or sensory adaptation (15, 17, 26). Specifically, butanone learning has been a major focus in the previous literature (8, 11, 26, 35). Butanone learning was first proposed to bidirectionally modulate chemotaxis index (11). However, learning and chemotaxis results can vary depending on the regime of butanone concentration used (8, 11, 36) and might produce variable chemotaxis indices across different repeated assay (8, 35). Therefore, later studies mostly focus on one of the two valances (appetitive vs naive, or aversive vs naive), with specific concentrations (12, 26). When appetitive and aversive learning are compared, past work has tested them at non-comparable odor concentration ranges (8, 26). In contrast, here we design a bivalent training protocol (aversive and appetitive) that shows clear effects even when tested in the same odor concentration range. To accomplish this our protocol is asymmetric with multiple exposures repeats aversive training and only a single exposure during appetitive training. We then conduct tests in an identical, controlled sensory environment enabled by our recently developed odor delivery system (23). The sensorimotor transformation altered by learning can then be directly compared through this approach (Figure 1).

Characterization of sensorimotor transformation in small animals such as C. elegans sheds light on the underlying neural computation (9, 15, 17) and navigation algorithms (37). Specifically, past studies have pinpointed neural circuits related to both biased random walk and weathervaning strategies for salt and thermal navigation (14, 15, 17). In contrast to only counting the number of worms ending up gradient, which is the classical calculation for chemotaxis index, we can better characterize sensorimotor transformation along the chemotaxis trajectories with the full tracking data. Moreover, as shown from simulation work, coarse measurements such as the chemotaxis index is often degenerate (22), since an agent can climb up gradient with different kinematics and strategies but result in similar chemotaxis index. It is therefore important to study how the worm respond to odor concentration along the navigation path.

Previous calculation for pirouette strategy (10) and weathervaning strategy (14, 17) rely on regressing the worm’s locomotion with respect to estimates of the sensory environment. This requires choosing a time window to calculated gradients, and defining behavior with hand-tuned parameters. Moreover, these regression approaches often omit quantification of uncertainty and noise. dPAW provides a framework to jointly fit navigation parameters and their uncertainties to data. Through statistical inference with dPAW, we find that learning might not only alter the baseline behavioral statistics (Figure 3) but also the temporal kernels for odor input. For instance, appetitive training sharpens the tangential concentration kernel (Figure 2). By contrast, classical approaches implicitly have static kernels, and therefore would have missed this important change. dPAW includes parameter that control the noise level of behavioral output, which is an advantage over past works (14, 22). Our finding shows that learning alters not only the sensing mechanism but also the behavioral statistics, which is consistent with recent work showing that starvation or neuromodulation can alter multiple behavioral traits (38, 39). Future work is needed to pinpoint the source of stochasticity, such as noise in the sensory or motor circuits, and its potential functional roles in navigation and exploration (40).

Statistical modeling and inference methods have been widely adopted in computational neuroscience, particularly in the study of sensory encoding (41, 42). Recent work has extended similar frameworks to characterize sensorimotor transformation in small animals (28, 34). These experiments conduct optogenetic stimulation in odor sensing neurons to characterize neural dynamics (30) or behavioral output (26). While these results focus on the elements of odor navigation, the mapping to sensorimotor transformation during a navigation task is still less clear. To deliver olfactory impulses, experimenters often have to either construct transgenic strains to optogenetically stimulate the sensory neurons or develop apparatus to deliver controlled odor impulse, which are both relatively challenging. In our work, we show that by applying statistical inference directly to navigational trajectories, the noninvasive method can indeed qualitatively recover sensorimotor transformation upon optogenetic perturbation (Figure 5). The result also shows that the variability in optogenetic response can be predicted by sensing kernels inferred from navigation across odor landscapes.

As shown in multiple studies (43–45), the result of associative learning is often heterogeneous across animals or non-stationary in time. Our result shows an opposite effect, where naive worms are more variable and the learning better “synchronizes” behavioral strategies across worms. Surprisingly, we further find that the variation across optogenetic responses in naive worms can in part be explained by the context in an odor environment, up or down the gradient. While recent work show population representation of odor stimuli in worms (25), our result shows that the AWC pathway is sufficient to capitulate learning effects and is causally altering navigation behavior.

The biophysics of the AWC^ON^ neuron (30, 32) and its circuit connection (8, 26, 31) has been studied in detail. Importantly, many connections to the first layer of interneurons are shared with salt sensing neurons ASER/L and thermal sensing neuron AFD. In the five interneurons we focused on, AIB has been shown to play a role in temperature learning (15). AIB, AIY, and AIZ all seem to be involved in learned salt chemotaxis strategies (17). Our findings agree with these results in showing that all three interneurons have non-zero and learning-dependent weights for both behavioral strategies. Through optogenetic control (46) and calcium imaging (33), AIY and RIA have been shown to support weathervaning behavior. Interestingly, we find that weathervaning in worms with ablated AIY and RIA increase their weathervaning index after appetitive and aversive trained conditions, respectively. This indicates that there might be compensatory effects from other circuits performing weathervaning in an experience-dependent manner. To date, the most comprehensive work on the neural circuits underlying butanone learning also shows that neural activity in steering neurons such as AIY and RIA are altered by learning (8). Our finding shows how they are deferentially modulated by learning, supporting that RIA is crucial for bidirectional learning. In addition, imaging results show that a population of sensory neurons are affected by learning (8) While we demonstrate that the driving the AWC^ON^ neuron is sufficient to recover learning-dependent behavior, analysis on the neural population level and its corresponding behavior would be an important extension.

To understand the underlying neural mechanisms that supports learned odor navigation in worms, potential future directions include imaging the neural population dynamics (8, 25) *during* odor navigation and study temporal progression of learning effects (47). Despite having high spatiotemporal resolution in neural recording, whole-brain imaging experiments are inherently low throughput. Typical neural imaging are on the order of 10 minutes and one worm at a time, oppose to chemotaxis with 100 worms with the same training procedure and recorded for 30 minutes in parallel. In the limit of finite data, our result show quantitative model comparisons and indicate that dPAW can better decode for training conditions with finite behavioral data. The fact that these behavioral variables are more informative about training conditions would guide our search for learning-dependent neural representations.

In this work we utilize a controlled odor environment and an innovative model to characterize learned odor navigation in worms. The combined approach of precisely delivered sensory stimuli, behavior quantification and navigational modeling continues to be a powerful approach (48–50) that can be generalized to other sensory modalities and species to study adaptive sensory navigation.

## Materials and Methods

### Worm strains and preparation.

We used N2 wild type worms for experiments shown in Figure 1 to Figure 4. The strain for optogenetics was integrated from the strain in (26). All strains were maintained on nematode growth medial (NGM) agar plates and fed with OP50 food. For interneuron perturbation, we integrated strains from (17) and used strains from (15). Detailed genetics and transgenic protocol are shown in supplementary information. Before chemotaxis experiments, we bleached batches of worms to synchronize the next generation. For optogenetic strains, worms were plated on 1ml OP50 food with 10μl all-trans retinal at L1 stage. For interneuron perturbation, miniSOG strains were treated with 2.16 mW/mm^2^ 450 nm blue light at 1 Hz for 30 minutes at L1 stage.

### Olfactory learning protocol.

Synchronized young adult worms were removed from food and washed three times with S. Basal solution (9). Appetitive trained worms were suspended in 10ml of S. Basal solution on a shaker to starve for 1 hour. After starvation, worms were placed on a 9 cm NGM agar plate with 1 ml of OP50 and 12 μl of butanone dropped on the lid. The butanone droplets were distributed across three agar plugs and the petri dish is sealed with parafilm. Aversive trained worms were suspended in 10 ml of S Basal with 1 μl butanone added. The tube was sealed and placed on the shaker for 1 hour. We empirically found that aversive training does not work without repetition, so we interleaved the session by plating the worms back on food for 30 minutes, then repeating the training for three times (Figure 1a). Naive worms were removed from food and washed for three times before testing directly. More details for the protocol and empirical observations are included in supplementary information.

### Odor flow chamber setup and chemotaxis experiments.

Following the different training protocols, worms were tested in the same odor flow chamber. This odor flow chamber enables continuous control through odorized air flow and monitor odor concentration through metal-oxide-based sensor arrays. Given a fixed flow parameter, we measured the steady-state odor landscape across the two-dimensional sensor array. This odor landscape was then used to infer odor concentration the worm experiences as it navigates along the same stationary odor environment. The assembling details of this apparatus is reported in (23). For chemotaxis experiments, we used 1.6% agar with salt content matching S. Basal in a 9 cm square plate. We used 11 mM butanone dissolved in water as the odor reservoir and moisturized clean air as the background flow. The background airflow is 400ml/min and the butanone odor flow is 33–35ml/min. After the pre-equilibration protocol (23) that brings the agar plate to steady-state in the odor environment, 50–100 worms were placed in the middle of agar plate and dried with kimwipes. Each chemotaxis sessions were record for 30 minutes.

### Behavioral imaging and optical setup.

Worm navigation behavior were recorded with a CMOS camera fixed above the odor flow chamber. Each image is 2500 × 3000 pixels, with each pixel as 32μm, and acquired at 14 Hz. Images were captured with custom written Labview program and analyzed with Matlab scripts (23). Worms were illuminated with 850 nm LED strip surrounding and above the agar plate. For optogenetic experiments, a long-pass filter is fixed in front of the camera lens to block the 525 nm stimuli. Three 525 nm LEDs were fixed on top of the flow chamber to deliver stimulation. The intensity was calibrated in the field of view with a photometer, with $85\;\mu W/{mm}^{2}$ for each uniform light impulse. Each pulse last for 5 seconds and were delivered every 30 seconds. Here we analyzed 1220–3060 pulses delivered to worms treated with retinal in each training conditions.

### Behavioral analysis and dPAW inference.

With the behavioral analysis pipeline (23), we tracked the location of the worm’s centroid and fit a centerline to its posture. We smoothed the navigation trajectories with a third-order polynomial with a 0.5 second time window. We removed trajectories that are shorted than 1 minute or have displacement less than 3 mm across the recordings. In addition, trajectories that started above 70% for the maximum odor concentration were also removed to prevent double-counting worms that have made it up gradient. This results in 270–1,140 animal hours of chemotaxis trajectory per training condition for model fitting. For each processes navigation trajectories, we computed the displacement vectors every 5 time bins (5/14 seconds) and compute the angle between consecutive vectors to obtain $d\theta_{t}$. We computed the odor concentration it passes through using the two-dimensional odor landscape measured in the flow chamber $C_{t}$. The perpendicular concentration difference $dC^{⊥}$ is calculated with unit vectors that are orthogonal to each displacement vector. We also recorded the length of each displacement vector to form the empirical speed distribution.

With the ensembles of trajectories, we fit dPAW by maximizing the log-likelihood:

[6] $$\text{argmax}\Sigma_{n}^{N}\Sigma_{t}^{T}\text{log}P\left(d\theta_{n,t+1}|C_{n,:t},dC_{n,:t}^{⊥},d\theta_{n,:t}\right)-\lambda {\left|K_{C}\right|}^{2}$$

where N is the number of trajectories, T is the time steps, and λ is the regularization term. To imposes smoothness on kernels, $K_{C}$ is parameterized with 4 raised-cosine basis function, $K_{dc^{⊥}}$ and $K_{h}$ are parameterized with an exponential form. Optimization was performed with constrained optimization in Matlab, where the constrains are positivity of the precision parameters and sigmoid probabilities. We show that the inference procedure works with simulated data and recovers the ground truth parameters. Uncertainty about the inferred parameters are characterized by numerically computing the Hessian of the log-likelihood function. For model-based decoding, we perform 7-fold cross validation with all measured trajectories. To test with finite data, we subsample 10 ensembles of trajectories to test for performance in Figure 4.

To generate chemotaxis trajectories from inferred parameters shown in Figure 2, we conduct agent-based simulation by measuring concentration in the same odor landscape and drawing angular change $d\theta_{t}$ from dPAW. We simulate two-dimensional navigation trajectories with:

[7] $$x_{t+1}=x_{t}+v_{t}cos\left(\theta_{t}\right)$$

[8] $$y_{t+1}=y_{t}+v_{t}\text{sin}\left(\theta_{t}\right)$$

[9] $$\theta_{t+1}=\theta_{t}+d\theta_{t}$$

where $v_{t}$ is speed drawn from Gaussian fit to the empirical distribution.

For information rate (Fig. 3) and model companions (Fig. 4), we construct null models to compare with dPAW. The random walk model has similar statistical structure for behavior but is independent to odor concentration:

[10] $$P\left(d\theta_{t}\right)=P\left(\beta =1\right)P_{pr}\left(d\theta \right)+P\left(\beta =0\right)P_{\text{run}}\left(d\theta \right)$$

Note that different from dPAW, the turning probability is time-independent, so β does not have a time subscript t. The pirouette behavior has the similar sharp angle, but now weathervaning is changed to “runs” that have zero-mean and do not take perpendicular concentration change into account. This model was fitted to the same ensemble of trajectories, and the log-likelihood difference between APAW and this model is normalized by log(2) per time to compute the bit rate. We also used this model to compute behavior-only model prediction in figure 4. The chemotaxis model is formulated with a binomial distribution with expected fraction of track going up gradient $\overset{ˆ}{p}$. The estimation for chemotaxis index is then $2\overset{ˆ}{p}-1$. Lastly, the concentration change model takes $C_{\text{final}\;}-C_{\text{initial}\;}$ for all tracks and uses naive Bayes classifier for prediction.

## Supplementary Material

1

## Significance Statement

Learning is generic across species of all scales. How does the brain flexibly learn to produce complex behavior? We focus on C. elegans to study how navigation strategies depend on olfactory learning by utilizing precise experimental measurements and a novel statistical model. The fitted model shows that learning alters two known navigation strategies in worms and outperforms the classical chemotaxis metric in decoding learning dependencies. We discover modulation of context-dependent behavior and decrease in behavioral variability after learning. In addition, through perturbing individual interneurons, we find that most neurons’ contribution are distributed across strategies and are differentially altered by learning. We expect these flexible properties in behavioral and neural computation to be generalized to other species and behavior.
