# Olfactory learning alters navigation strategies and behavioral variability in C. elegans

**Published:** 2024-02-23

**Authors:** Kevin S. Chen, Anuj K. Sharma, Jonathan W. Pillow, Andrew M. Leifer

## Abstract

Animals adjust their behavioral response to sensory input adaptively depending on past experiences. The flexible brain computation is crucial for survival and is of great interest in neuroscience. The nematode C. elegans modulates its navigation behavior depending on the association of odor butanone with food (appetitive training) or starvation (aversive training), and will then climb up the butanone gradient or ignore it, respectively. However, the exact change in navigation strategy in response to learning is still unknown. Here we study the learned odor navigation in worms by combining precise experimental measurement and a novel descriptive model of navigation. Our model consists of two known navigation strategies in worms: biased random walk and weathervaning. We infer weights on these strategies by applying the model to worm navigation trajectories and the exact odor concentration it experiences. Compared to naive worms, appetitive trained worms up-regulate the biased random walk strategy, and aversive trained worms down-regulate the weathervaning strategy. The statistical model provides prediction with $>90 \%$ accuracy of the past training condition given navigation data, which outperforms the classical chemotaxis metric. We find that the behavioral variability is altered by learning, such that worms are less variable after training compared to naive ones. The model further predicts the learning-dependent response and variability under optogenetic perturbation of the olfactory neuron AWC$^\mathrm{ON}$. Lastly, we investigate neural circuits downstream from AWC$^\mathrm{ON}$ that are differentially recruited for learned odor-guided navigation. Together, we provide a new paradigm to quantify flexible navigation algorithms and pinpoint the underlying neural substrates.

