# Supplementary material for: Olfactory learning alters navigation strategies and behavioral variability in c. elegans
Source: ArXiv. 2023 Nov 13:arXiv:2311.07117v1. Preprint. [Version 1] (PMC10680870)
Supplement: Supplementary file 1 [file NIHPP2311.07117V1-supplement-1.pdf]

arXiv:2311.07117v1 [q-bio.NC] 13 Nov 2023

Supporting Information for

Olfactory learning alters navigation strategies and behavioral variability in *C. elegans*

Kevin S. Chen, Jonathan W. Pillow, and Andrew M. Leifer

Andrew M. Leifer.
E-mail: leifer@princeton.edu

This PDF file includes:

Supporting text
Figs. S1 to S10
Table S1
Legends for Dataset S1 to S2
SI References

Other supporting materials for this manuscript include the following:

Datasets S1 to S2

Supporting Information Text

1. Experiments with learned olfactory navigation

Odor deliver system. We used the odor flow delivery system and experimental protocols developed in (1) to measure chemotaxis trajectories after different training conditions. In short, this system incorporates controlled odorized airflow and continuously measures odor concentration along the boundary of an agar plate during animal experiments. We calibrated the array metal-oxide based sensors with a downstream photo-ionization detector (Figure S1a). The sensor array was used to measure the steady-state spatial profile before chemotaxis experiments (Figure S1b). During animal experiments, we swapped out sensors in the middle of the arena to place in worms on an agar plate and continued to measure the boundary condition to confirm that the odor landscape is controlled and stable across the arena (Figure S1c).

Olfactory learning protocol. The olfactory learning protocol was developed by modifying from previous literature (2–5). We found that significant aversive conditioning was particular difficult to achieve, in both our flow chamber or with traditional plate assays. We discovered that repeating the training step by three times makes the effect significantly different from naive conditions (Figure S2). With the repetition protocol, we also discovered that it is more efficient to training with liquid form, repeated starvation causes worms to craw into the agar plates and potentially biases the final selected worms.

2. Inference and model comparison

dPAW inference. We simulated behavioral time series from dPAW with a fixed set of ground-truth parameters and tested the performance of inference procedure. As we do not know how to simulate chemotaxis-like behavior a priori, we applied Gaussian random white noise for concentration time series C_t and $dC_t^{\perp}$. We confirmed that the ground truth parameters can be robustly recovered (Figure S3). The kernels, densities modelling the mixture of strategies, and the nonlinear functions could be identified through maximum likelihood method.

With dPAW fitted to experimental data, we confirmed that the inferred densities for head angles $d\theta$ matches the observed distribution (Figure S4a). Two mixtures of von Mises distributions can approximated the features observed from experiments. We noticed that the mismatch is largest in a range of intermediate angles, potentially related to other turning strategies previously reported in worms (6). Future work can append a third density to the same dPAW framework. In addition, we set a threshold at $d\theta = 150$ degrees as an empirical definition of a sharp turn. With this threshold, the estimated turning probability across three learning conditions show agreement between experimental observations and model predictions (Figure S4b).

Model comparison. We explored the importance of specific kernel parameters by computing the information rate and kinematics in models with ablated parameters. Across three learning conditions, the kernel for pirouette decision K_C contains significantly larger information content about the worm's behavior compared to the weathervaning kernel $K_{dC^{\perp}}$ (Figure S5a). For the angular history kernel K_h, we added this parameter during the construction of dPAW as we empirically found that it helps to capture the observed inter-turn-intervals. It has been reported that the worms produce sharp turns in bouts with specific time scales, and the original definition of a pirouette with respect to the time scale of multiple sharp turns close in time (7). We found that our model can generate tracks that have similar kinetics with the history kernel and without explicitly modeling state-transitions (Figure S5b).

We sub-sampled chemotaxis trajectories for training dPAW and test on held-out trajectories. We confirmed that the subsets of trajectories are quite representative, by showing the kernels K_C fitted to different subsets of trajectories (Figure S6). At a large data set limit, the variability of these kernels inferred from sub-sampling should agree with the standard deviation estimated with the Hessian of the log-likelihood function. In the context of decoding, we explored why the concentration difference ΔC along each track does not provide significant information to decode learning conditions. We found that the histogram shows limited separation in three groups (Figure S7) and the distribution does not change significantly as we include more trajectories.

3. Optogenetic perturbation

Additional analysis with odor and optogenetic signals. We sub-sampled from trajectories recorded from chemotaxis with optogenetic perturbation. For each given ensemble of trajectories, we fit the statistical model for pirouette probability (equation 5) and compute the norm of kernels K_{opto} and K_{odor}. We re-sampled ensemble of 100 trajectories from 300-1000 trajectories across three learning conditions (Figure S8a). The resulting scatter plot shows that appetitive trained worms have a relatively clustered large weight on odor and is independent to the response to optogenetic impulse. The naive worms have weights on odor and optogenetic input that are weakly but significantly correlated. This means that subset of trajectories that vary and have less weight on odor signal would also have less optogenetic response. Lastly, aversive worm have clustered small weight on odor and is uncorrelated to the optogenetic input. Together, the sub-sampling result is consistent with the observation in Figure 5 and supports the finding that tracks going down gradient (less weight on odor input) also respond less to optogenetic input.

Mapping the behavior-triggered average with optogenetics. While we fit response kernels to impulse of optogenetic signal, a convention way to compute these response functions is to compute the behavior-triggered average (BTA) under white noise stimuli (Figure S8b,c). The white noise stimulus is drawn from $\mathcal{N}(30, 30)$ (μW/mm^2) at 14Hz with 0.5s correlation time and

bounded between 0-60 μW/mm^2. Given white noise input, one can map out a linear-nonlinear model for behavior transitions with similar methods applied to touch sensation in worms (8). We carried out separate experiments without the odor landscape, mapping BTAs after three training conditions. The resulting BTAs are bidirectionally modulated by learning, compared to naive condition, which is consistent with the odor kernels K_{odor} inferred from chemotaxis trajectories along odor gradient. We did not combine white noise stimuli with the full chemotaxis experiments since the effect of continuous random input during navigation might be harder to characterized. The exact kernel shape of K_{opto} does not match BTA, potentially due to worms navigating in the background of odor landscape versus an empty plate, or due to the difference in sensorimotor transformation upon a transient light pulse versus continuous noise. Nonetheless, the bidirectional modulation across learning conditions and the response time scale of the measured BTA corroborates conclusions drawn from optogenetic perturbation during chemotaxis in Figure 5.

4. Chemotaxis in worms with disrupted interneurons

The transgenic strains with miniSOG come from (6). For the strain with AIB(-), we integrate from strain in (9) and have previously reported it (1). AIB(-) strain over-expresses active potassium channel to down-regulate the interneuron. While this disruption mechanism is slightly different from ablation through miniSOG method, we argue that down-regulation methods mostly have agreeable effects, as reported in (6) where they compared multiple ablation and inhibition methods. The list of strains used in this work are included in the supplementary table.

Metric and statistical tests. We measured chemotaxis behavior in transgenic worms with disrupted interneurons. We fit dPAW to each train after three different training conditions. Due to certain strains that have significantly less locomotion after transgenic manipulation, we computed the concentration weighted chemotaxis index: $(N_{up}\Delta C_{up} - N_{down}\Delta C_{down})/(N_{up}\Delta C_{up} + N_{down}\Delta C_{down})$, where N is the number of tracks going up or down gradient and ΔC is computed for every track. We find the maximum and minimum concentration across the full observed odor landscape, then define $\Delta C_{up} = C_{max} - C_{final}$ for tracks going up gradient and $\Delta C_{down} = C_{final} - C_{min}$ for tracks going down gradient, with ending concentration C_{final}. We found that this weighted chemotaxis index better reflects the chemotaxis performance as it takes into account the displacement across odor concentration.

To conduct statistical tests between indices in Figure 6, we re-sampled from 200-500 chemotaxis trajectories in each conditions. For the weighted chemotaxis index, we sampled 50 tracks for 20 times to compute the standard deviation from mean. For biased random walk strategy, we computed $|K_C|/std(C_t)$ to quantify the weights on concentration C and normalized by the standard deviation of the time series itself to compare across strains that experience different concentration inputs. For weathervaning strategy, we computed $std(K_{dC^\perp} * dC^\perp_{:t})$ to characterize how much the worm weights and corrects for the heading in response to $dC^\perp$. We again re-sampled from the subset of trajectories 20 times to compute the standard deviation of the metrics and conduct t-test between the transgenic strain and wild type (Figure S10). Another method of checking on variability is to sample from the posteriors of these kernels, with Gaussian approximation around the maximum likelihood estimate shown in Figure 2 b,c. Sampling from the posterior estimates did not change the conclusion from sampling trajectories, so we only report the result with the later procedure.

Additional observations. We noticed that the index for navigation strategies and the chemotaxis performance don't necessarily "add up" in certain strains. For instance, AIA(-) and AIB(-) worms that are appetitive trained have the same weighted CI as wild-type, but their BRW and WV index are significantly altered. Similar patterns have also been shown in past literature (10). It is possible that worms can adaptively employ different strategies to reach similar chemotaxis performance. For instance, we qualitatively observed that AIB(-) worms have less sharp turns and have curving trajectories in space, but still end up moving up gradient (Figure S9). In contrast, AIY(-) worms have high rate of sharp turns and move up gradient with non-smooth trajectories. Future work with models that have alternative strategies or more hierarchical structures are needed to characterize the complexity of these chemotaxis patterns.

While effectively capturing learning effects in our work, dPAW is a simplified description of worm chemotaxis. Specific shallow turns (6) and the varying speed of movement are neglected in dPAW. It is also possible that the worms use a richer combination of strategies to perform chemotaxis that cannot be reflected in weights on individual behavioral strategies. Another main assumption in dPAW, as well as other chemotaxis characterization in previous work (7, 10), is that the behavioral responses are stationary through time. It is possible that worms can respond differently in response to different local concentration gradients. Future work is needed to characterize behavioral dynamics during chemotaxis.

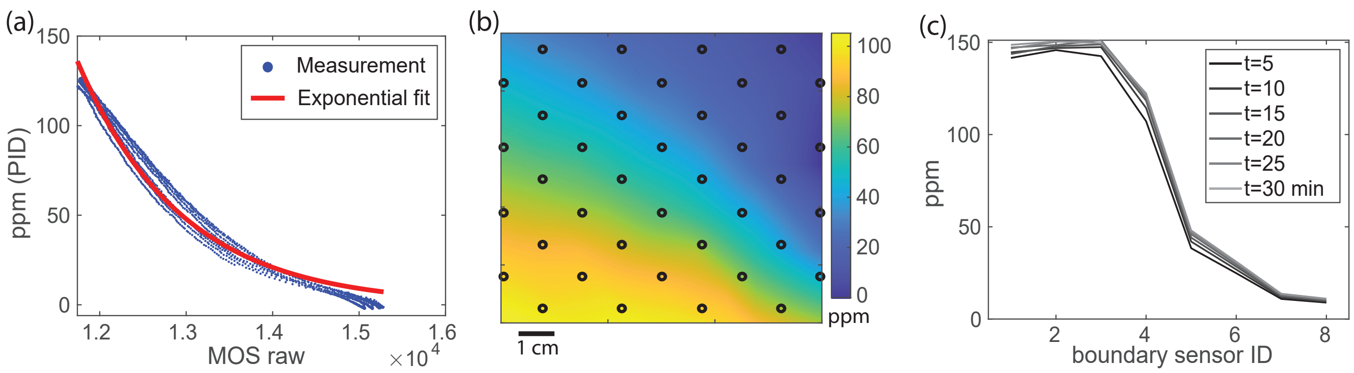

Fig. S1. Continuous controlling and measuring the odor environment. **(a)** Calibrating the metal-oxide sensors (MOS) with a downstream photoionization detector (PID). **(b)** Overlaid sensor array position (black circles) with the interpolated odor landscape. **(c)** Readout from a row of boundary sensors during animal experiments. The profile through time is shown in grade-scales

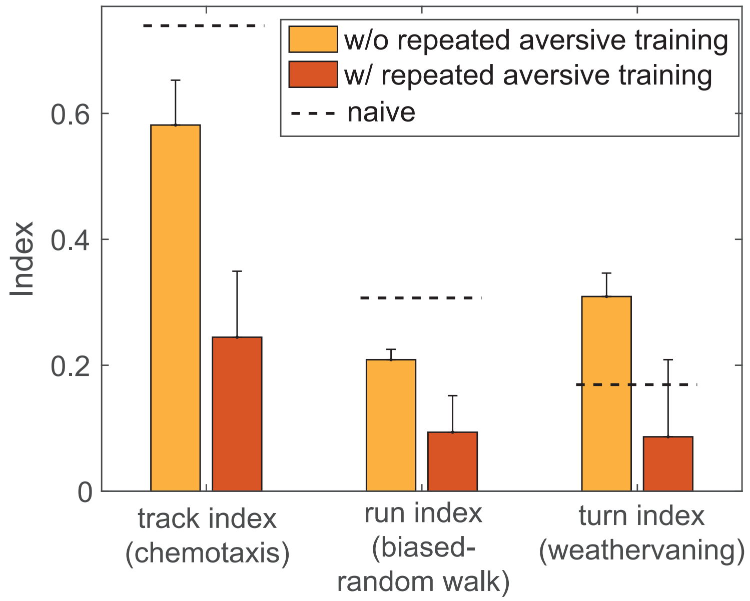

Fig. S2. Effects of repeated aversive training. We compare conditions without repeated aversive training (single trial) and with repetition (three times). The chemotaxis performance and the usage of two strategies are quantified and compared to naive condition (dash line). Error bar shows standard error of mean from 7 plates.

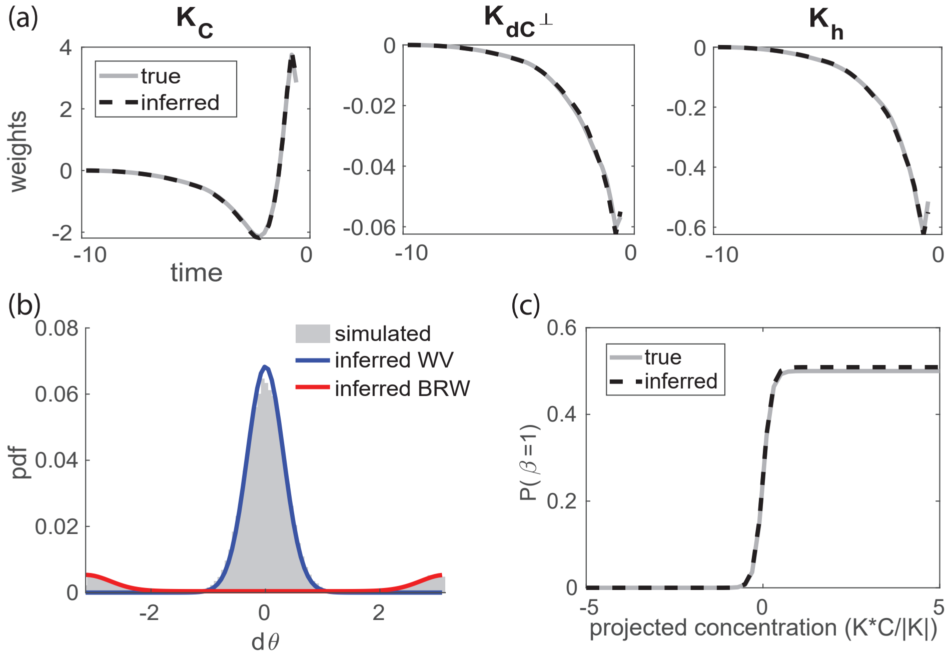

Fig. S3. dPAW inference with ground-truth parameters. **(a)** Comparing true and inferred kernels in dPAW. **(b)** The $d\theta$ distribution simulated from ground truth parameters overlaid with the inferred densities that come from a mixture of two strategies, weathervaning (WV) and biased random walk (BRW). **(c)** The sigmoid-like function for pirouette decision as a function of concentration projected onto the normalized kernel K_C

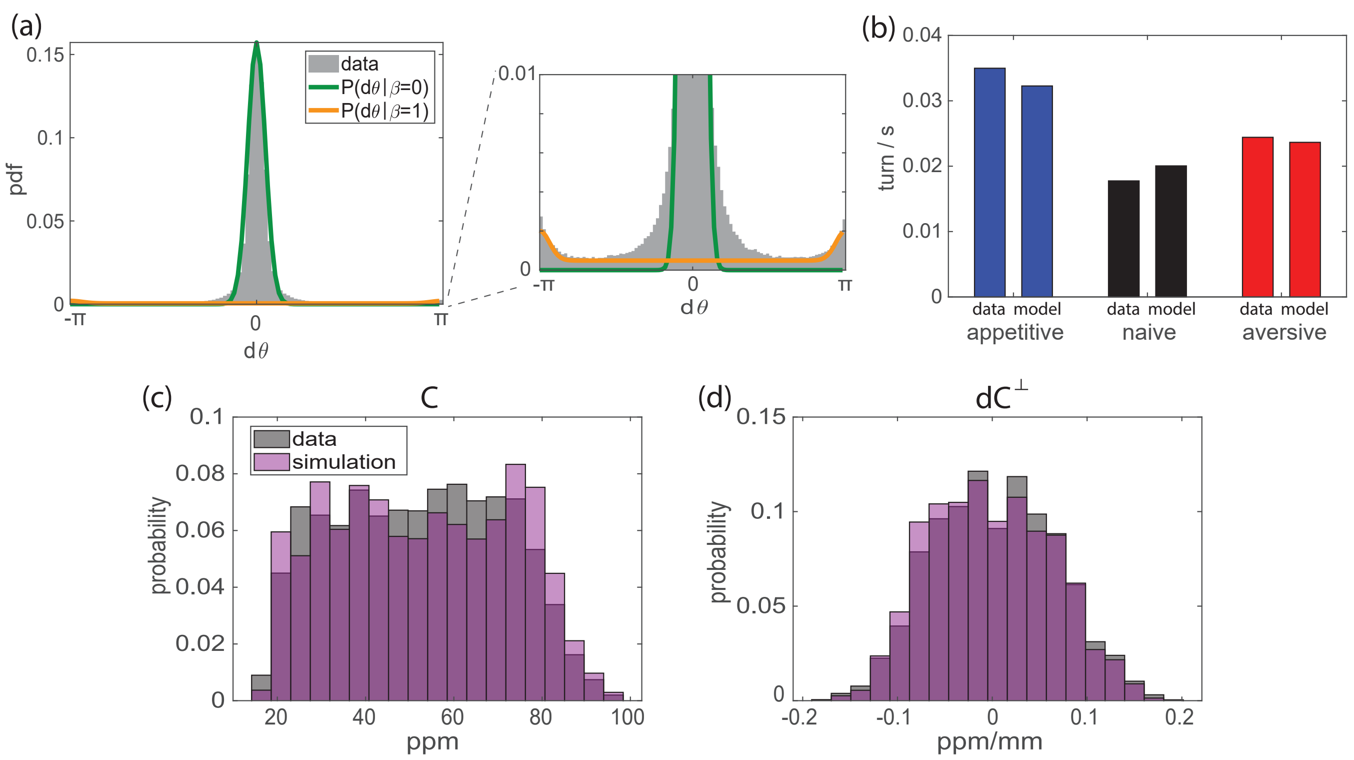

Fig. S4. Capturing observed statistics with dPAW. **(a)** The observed $d\theta$ distribution overlaid with the inferred mixture of two strategies. Zoom-in for a lower probability regime is shown on the right to visualize the pirouette angles ($\beta = 1$). **(b)** The empirical turn rate ($d\theta > 150$) in data and predicted by the inferred dPAW. The empirical and simulated concentration distribution **(c)** and difference of perpendicular concentration **(d)** are shown.

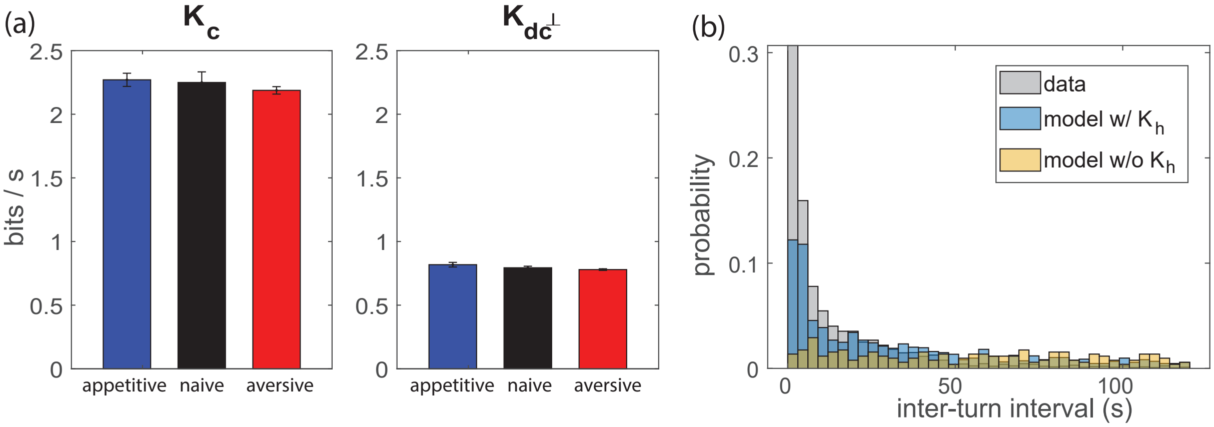

Fig. S5. Effects of kernels in dPAW. **(a)** The information rate of dPAW compared to the model with ablated kernel K_C. Error bar shows standard deviation across 7 fitted ensembles of trajectories. **(b)** same as (a) but with ablated kernel $K_{dC^\perp}$. **(c)** The distribution of inter-turn-intervals observed from data, trajectories simulated from the inferred dPAW with or without kernel K_h.

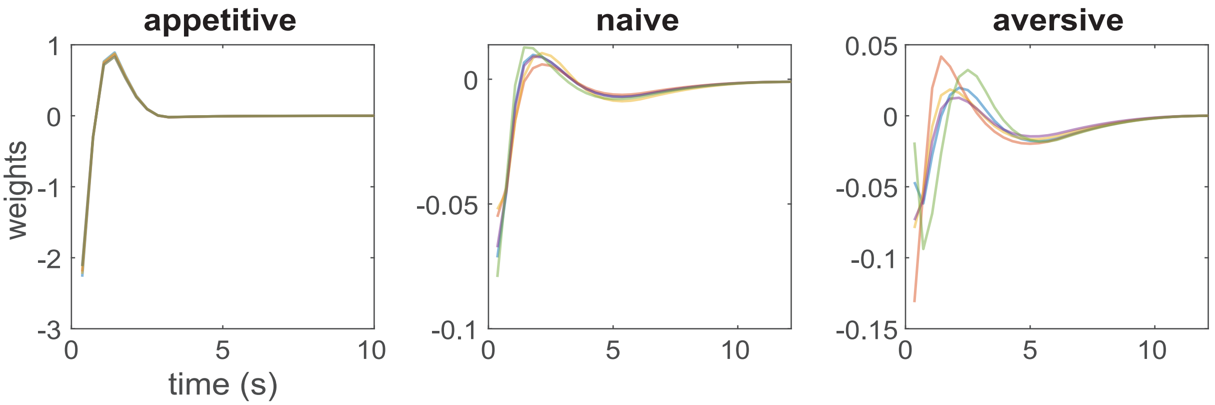

Fig. S6. The inferred kernels K_C from 5 sub-sampled ensembles of trajectories across three different training conditions.

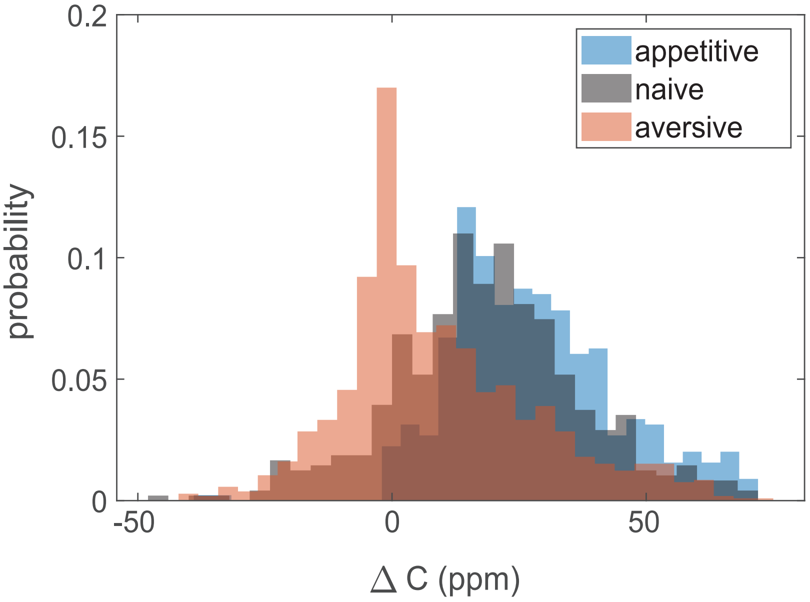

Fig. S7. Histogram of the odor concentration change ΔC along all trajectories observed after three different training conditions.

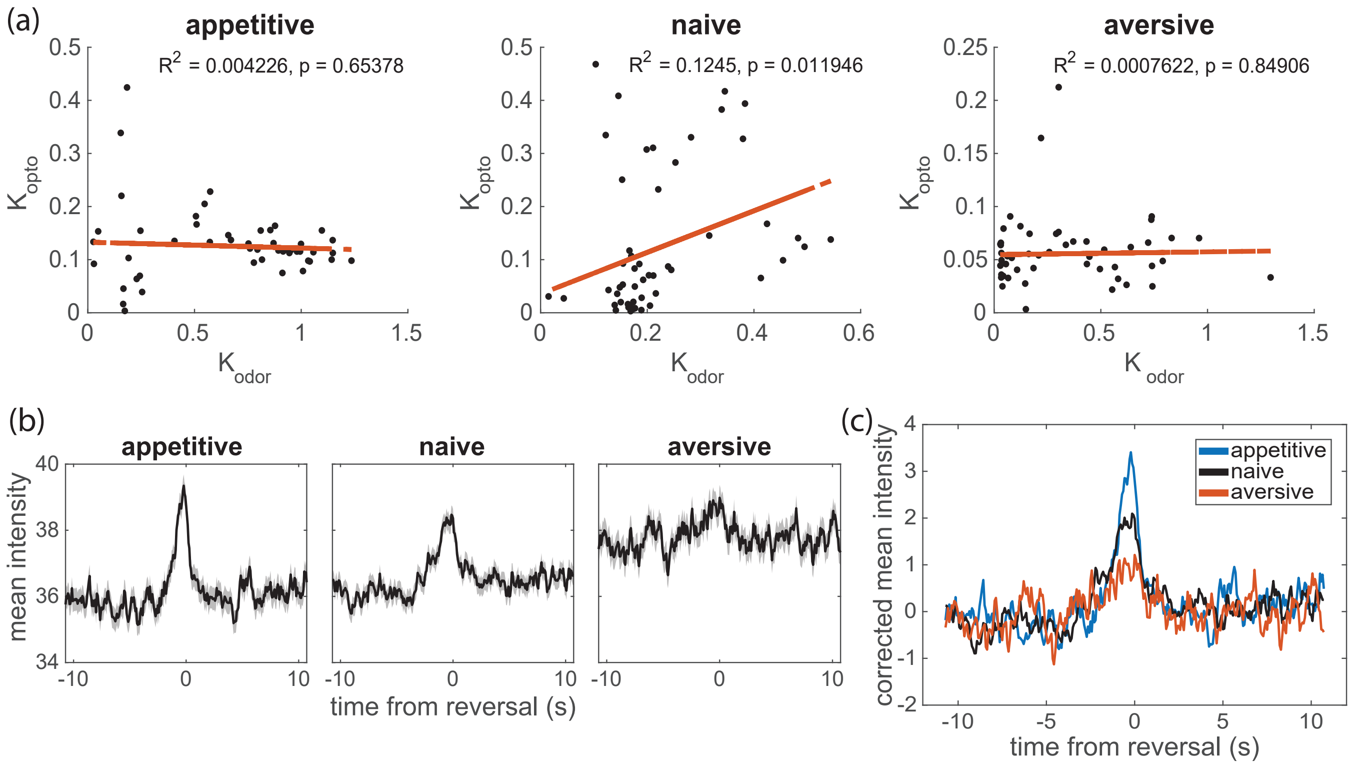

Fig. S8. Additional analysis with optogenetic perturbations. **(a)** Comparing the norm of kernels K_{opto} and K_{odor} from sub-sampled trajectory ensembles. The scattered samples show significant correlation in naive condition. **(b)** Behavioral triggered average (BTA) computed from Gaussian white noise stimuli after three training conditions. The shaded area shows standard error of mean around the mean intensity (μW/mm^2). **(c)** Comparing BTA from (b) after baseline subtraction for three training conditions.

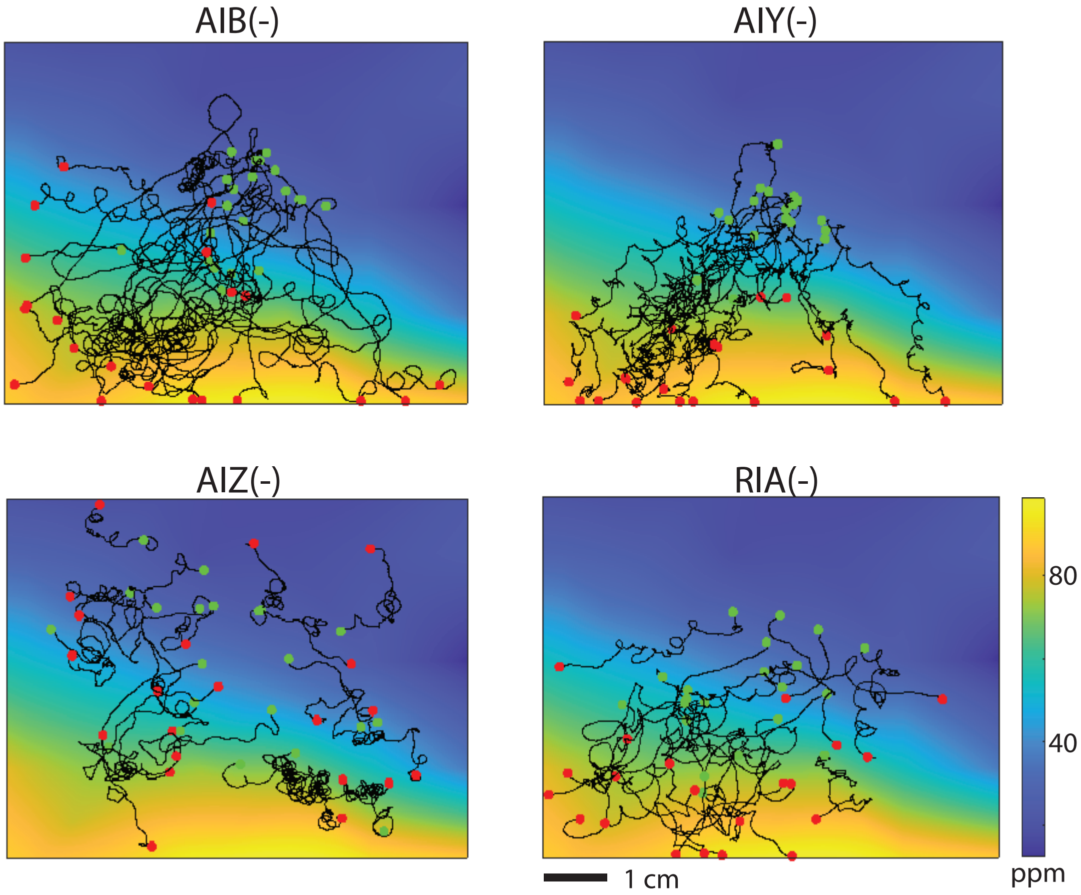

Fig. S9. Example chemotaxis trajectories from worms with disrupted interneurons. Here we show behavior after appetitive training in four different strains. The starting point of example tracks are shown in green and the terminal position is shown in red.

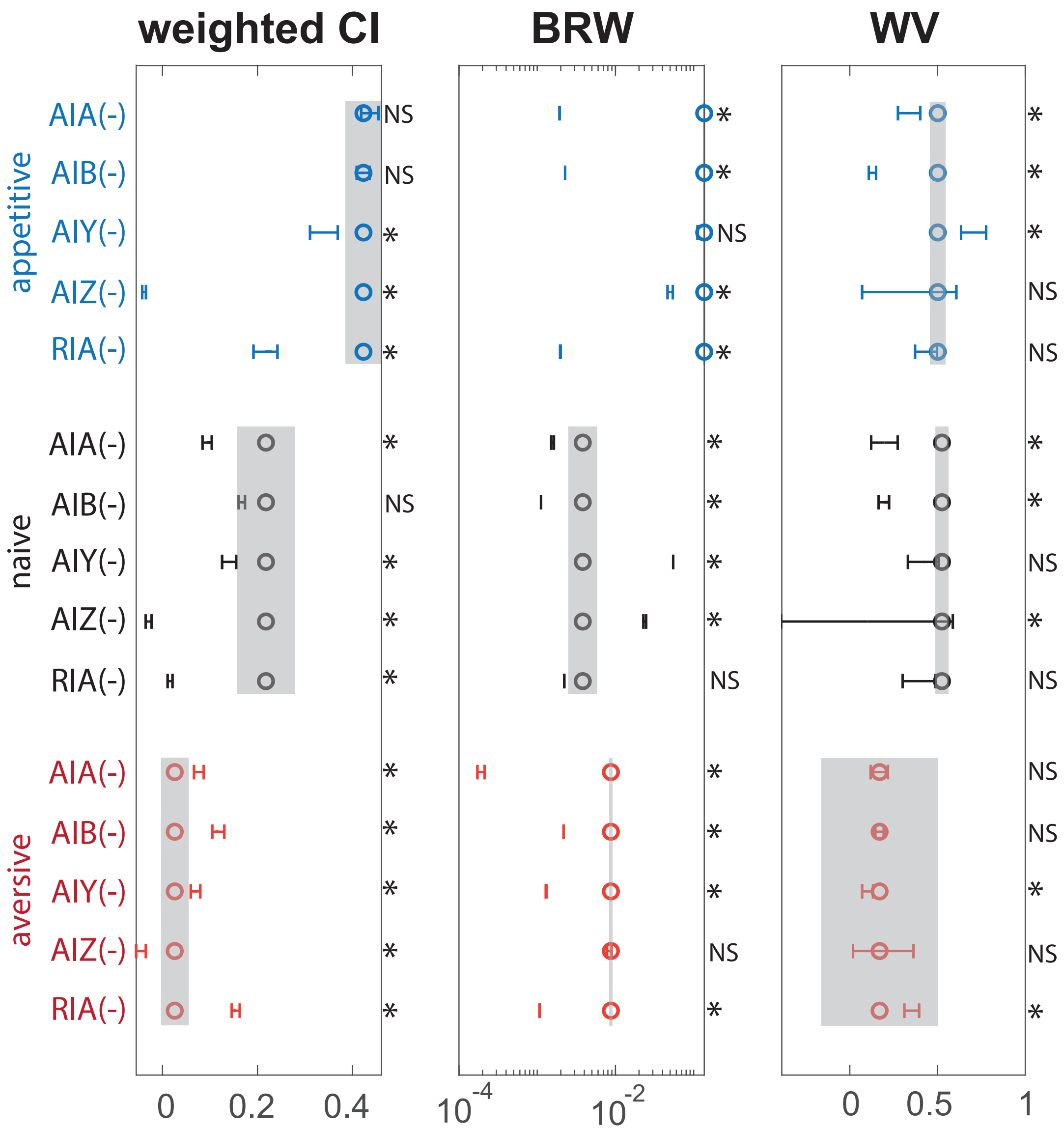

Fig. S10. Statistical tests for learned chemotaxis and behavioral strategies across worms with disrupted interneurons. For wild type worms, the mean values are shown in circle and the standard deviation is indicated with the shaded area. Across strains and learning conditions (color-coded), the mean and standard deviation are plotted for comparison to wild-type. The result from t-test ($*$ for $p < 0.01$ or non-significance, NS) is shown on the right side.

Table S1. Table for worm strains

Strain	Designation	Source or reference	Additional information
Wild-type *C. elegans*	N2	CGC	
ChR2 expressed in AWC^{ON}	AML61.1	this work	integrated from strain in (2)
AIB(-) activated potassium channel	AML580	(1)	integrated from strain in (9)
AIA(-) miniSOG	IK3240	(6)	IK strains are a gift from I. Mori
AIY(-) miniSOG	IK2962	(6)	
AIZ(-) miniSOG	IK3241	(6)	
RIA(-) miniSOG	IK3289	(6)	

SI Dataset S1 (Data)

Data to generate figures in this work would be posted on figshare website upon paper submition.

SI Dataset S2 (Code)

Code used for analysis and to generate figures are on github: https://github.com/Kevin-Sean-Chen/Chemotaxis_function. This repository would be updated upon publication.

References

1. KS Chen, R Wu, MH Gershow, AM Leifer, Continuous odor profile monitoring to study olfactory navigation in small animals. *Elife* **12** (2023).
2. CE Cho, C Brueggemann, ND L'Etoile, CI Bargmann, Parallel encoding of sensory history and behavioral preference during caenorhabditis elegans olfactory learning. *eLife Sci.* **5**, e14000 (2016).
3. C Pritz, et al., Principles for coding associative memories in a compact neural network. *Elife* **12** (2023).
4. A Kauffman, et al., C. elegans positive butanone learning, short-term, and long-term associative memory assays. *J. Vis. Exp.* (2011).
5. I Torayama, T Ishihara, I Katsura, Caenorhabditis elegans integrates the signals of butanone and food to enhance chemotaxis to butanone. *J. Neurosci.* **27**, 741–750 (2007).
6. M Ikeda, et al., Context-dependent operation of neural circuits underlies a navigation behavior in caenorhabditis elegans. *Proc. Natl. Acad. Sci. U. S. A.* **117**, 6178–6188 (2020).
7. JT Pierce-Shimomura, TM Morse, SR Lockery, The fundamental role of pirouettes in caenorhabditis elegans chemotaxis. *J. Neurosci.* **19**, 9557–9569 (1999).
8. M Liu, AK Sharma, JW Shaevitz, AM Leifer, Temporal processing and context dependency in caenorhabditis elegans response to mechanosensation. *Elife* **7** (2018).
9. L Luo, et al., Dynamic encoding of perception, memory, and movement in a c. elegans chemotaxis circuit. *Neuron* **82**, 1115–1128 (2014).
10. Y Iino, K Yoshida, Parallel use of two behavioral mechanisms for chemotaxis in caenorhabditis elegans. *J. Neurosci.* **29**, 5370–5380 (2009).
